# CorrelaGenes: a new tool for the interpretation of the human transcriptome

**DOI:** 10.1186/1471-2105-15-S1-S6

**Published:** 2014-01-10

**Authors:** Paolo Cremaschi, Sergio Rovida, Lucia Sacchi, Antonella Lisa, Francesca Calvi, Alessandra Montecucco, Giuseppe Biamonti, Silvia Bione, Gianni Sacchi

**Affiliations:** 1 Institute of Molecular Genetics, National Research Council, 27100 Pavia, Italy; 2 Institute of Applied Mathematics and Information Technology "Enrico Magenes", National Research Council, 27100 Pavia, Italy; 3 Dipartimento di Ingegneria Industriale e dell'Informazione, University of Pavia, 27100 Pavia, Italy

## Abstract

**Background:**

The amount of gene expression data available in public repositories has grown exponentially in the last years, now requiring new data mining tools to transform them in information easily accessible to biologists.

**Results:**

By exploiting expression data publicly available in the Gene Expression Omnibus (GEO) database, we developed a new bioinformatics tool aimed at the identification of genes whose expression appeared simultaneously altered in different experimental conditions, thus suggesting co-regulation or coordinated action in the same biological process. To accomplish this task, we used the 978 human GEO Curated DataSets and we manually performed the selection of 2,109 pair-wise comparisons based on their biological rationale. The lists of differentially expressed genes, obtained from the selected comparisons, were stored in a PostgreSQL database and used as data source for the CorrelaGenes tool. Our application uses a customized Association Rule Mining (ARM) algorithm to identify sets of genes showing expression profiles correlated with a gene of interest. The significance of the correlation is measured coupling the Lift, a well-known standard ARM index, and the χ^2 ^p value. The manually curated selection of the comparisons and the developed algorithm constitute a new approach in the field of gene expression profiling studies. Simulation performed on 100 randomly selected target genes allowed us to evaluate the efficiency of the procedure and to obtain preliminary data demonstrating the consistency of the results.

**Conclusions:**

The preliminary results of the simulation showed how CorrelaGenes could contribute to the characterization of molecular pathways and biological processes integrating data obtained from other applications and available in public repositories.

## Background

The comprehension of the molecular mechanisms involved in the physiology of human cells requires the development of new bioinformatics and biostatistics tools able to integrate and interpret the huge amount of data derived from different kinds of genome-wide approaches. The interpretation of the transcriptional state of the cell and its alterations in specific experimental or pathological conditions is today of particular interest and several technologies have been developed to identify and quantify the entire set of cellular transcripts. As a consequence, the amount of gene expression data available in public repositories has grown exponentially in the last years, now requiring new data mining tools to extract biologically relevant information.

Many databases of genome-wide expression data are today publicly available. Gene Expression Omnibus (GEO) developed at NCBI [1] and ArrayExpress developed at EBI [2] are the two main international repositories where about 45% of microarray published studies has been deposited [3]. A standardized system for reporting microarray results (Minimum Information About a Microarray Experiment, MIAME) [4] has been developed in order to facilitate the sharing of high-throughput data among scientists. These improvements made it possible to develop a variety of added-value databases that process and analyze expression data in order to answer to specific biological questions [5]. Different methods have been exploited to combine data from different sources in meta-analysis studies to reveal new aspects of biological processes even if data heterogeneity represents a challenge. Many procedures were developed in recent years to overcome this issue resulting in the availability of different bioinformatics tools. For example, the Oncomine application [6] considers gene expression datasets related to the tumorigenic transformation and the PubLiME tool [7] bases its analysis mainly on gene signatures. In COXPRESdb [8] a homogeneous set of data was selected from two human platforms and it was compared to expression data from different organisms. Each of these solutions offers a view of the whole set of expression data from a different perspective. For this reason, despite the availability of several databases and analysis tools, new bioinformatics approaches to query the increasing amount of expression data are still required.

In this context we developed CorrelaGenes, a new bioinformatics tool exploiting GEO expression data to provide new insights about the pathways in which a gene of interest could be involved [9]. CorrelaGenes is aimed at identifying lists of genes potentially correlated to a gene of interest. This is accomplished through a cross-sectional analysis among data from different microarray studies with the ultimate goal of detecting those genes showing modulation of their expression in a significant number of different conditions. The CorrelaGenes tool implements a customized Association Rule Mining (ARM) algorithm and a set of indexes that allow the user to dynamically explore his target gene combining different input parameters.

In this paper we will describe the process of data elaboration from the GEO archive to the results output file that users could obtain through the web interface. We will also describe the indexes we implemented to achieve a reliable selection of genes correlated with the input target and their impact on a simulation on 100 randomly selected genes. Moreover, we will show preliminary data about the biological relevance of CorrelaGenes results and how the possibility to choose the type of gene expression alterations (i.e. up- or down-regulated or both) will help in the elucidation of molecular pathways and of their players.

## Implementation

**Figure 1:**
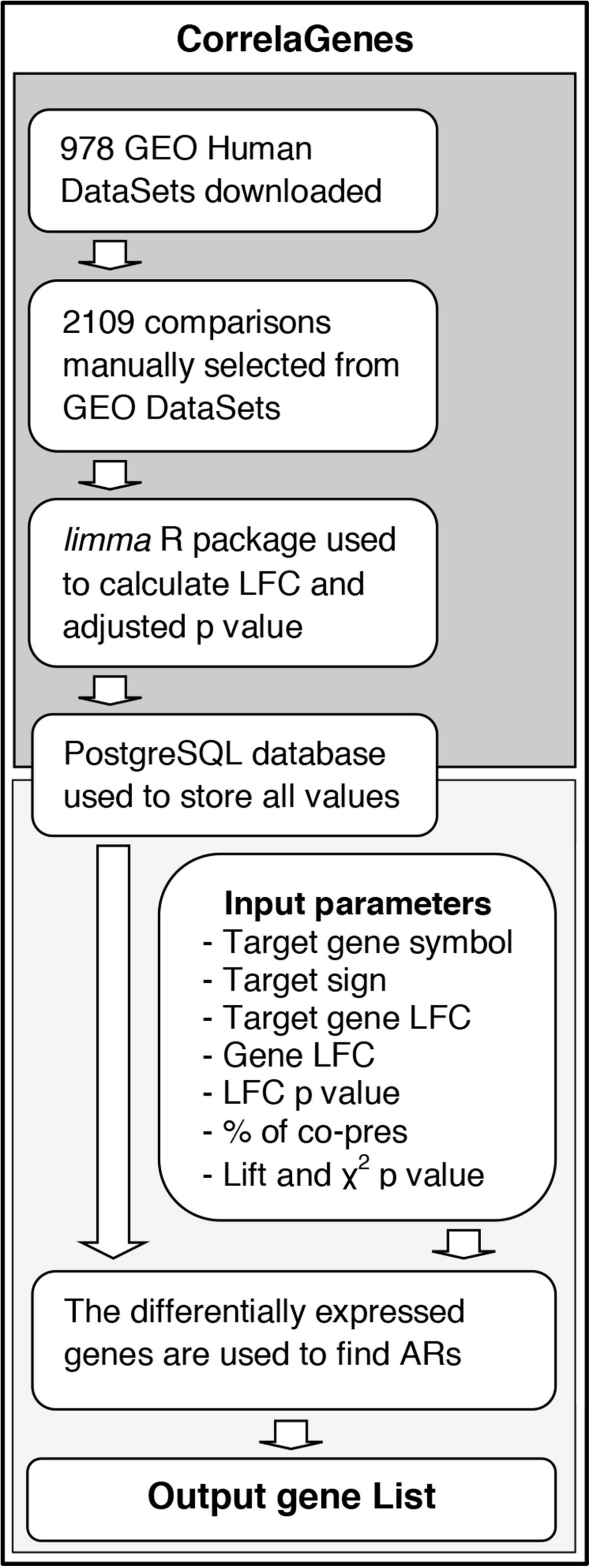
**Schematic representation of the CorrelaGenes workflow**.

CorrelaGenes was conceived to explore the biological role of a gene of interest selected by the user identifying a set of genes whose expression appeared altered in the same experimental or physiological state. The tool exploits GEO expression data and uses a customized Association Rule Mining algorithm for a cross-sectional analysis aimed at identifying those genes showing a coordinated modulation of their transcriptional profiles in different conditions. A schematic representation of the CorrelaGenes workflow is shown in Figure 1.

### Data pre-processing

**Figure 2:**
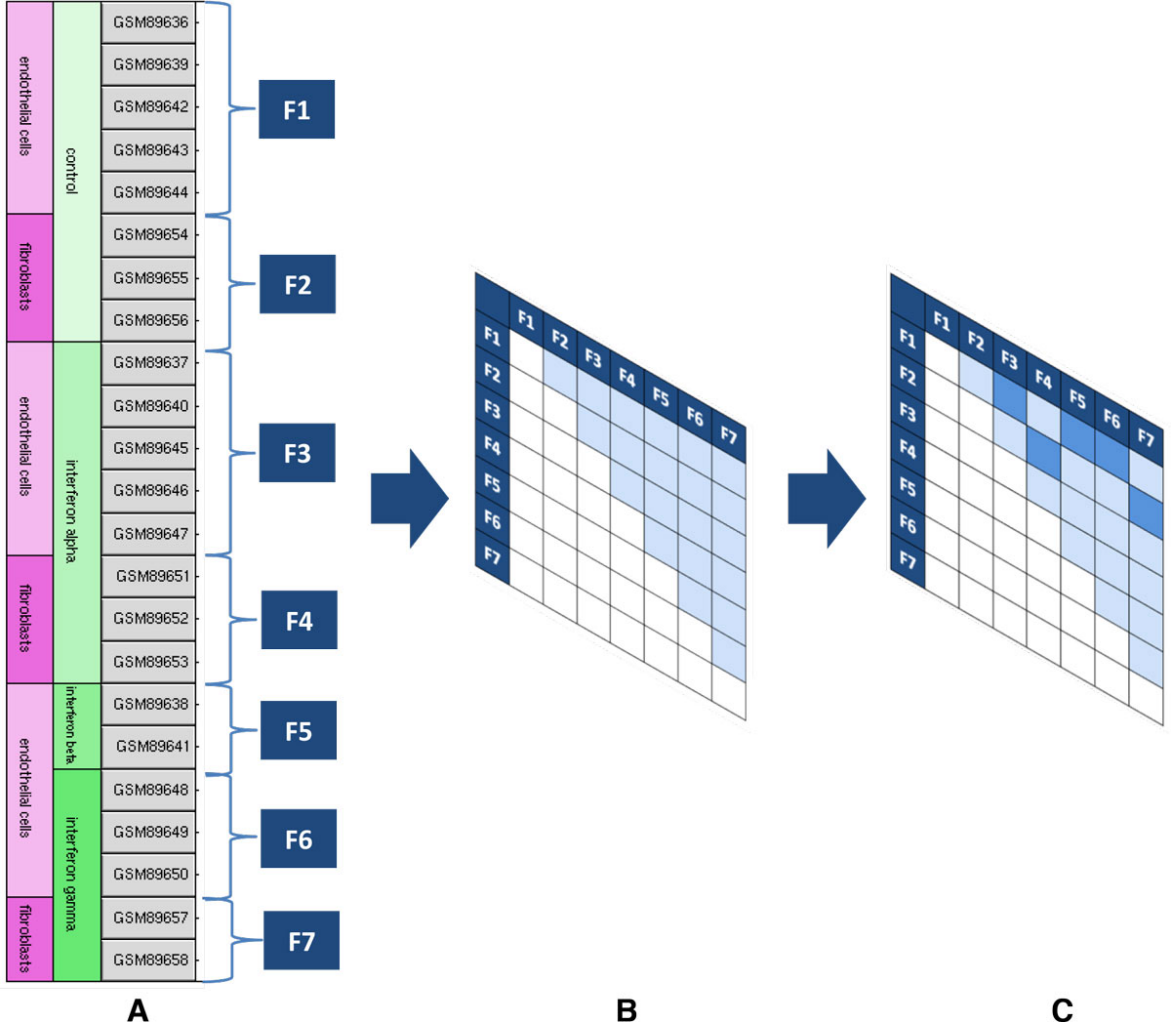
**Schematic representation of GDS2516 processing**. (A) Design of GDS2516 and experimental factor definition (F1 to F7). (B) Contrast matrix created with all groups versus group comparisons. In light blue are shown the 21 pair-wise comparisons. (C) Comparisons manually selected by the experts. In dark blue are shown the 5 comparisons selected.

The source of the expression data used by the CorrelaGenes tool was built from 978 human GEO Curated DataSets (GDS) downloaded from the GEO archive through the GEOquery 2.21.9 R/Bioconductor package [10]. The experimental design related to each GDS was used to group the intensity values measured for single sample sharing the same experimental factors (Figure 2A). Groups containing less than two samples were not suitable for subsequent analysis thus leading to discard a total of 261 GDS. The resulting groups were used to create a contrast matrix including all groups versus group comparisons (Figure 2B). A manually curated knowledge-based procedure was applied to select appropriate comparisons: as automatically generated matrices often include contrasts without a clear experimental meaning, a team of biologists defined a set of rules to extract those comparisons showing a strong biological rationale (Figure 2C). Figure 2 shows the procedure applied to GDS2516 (see also Additional File 1 for a more detailed description of the whole procedure). In this example a total of seven experimental factors were identified and used to create an all groups versus group contrast matrix of 21 comparisons among which only five were selected by the experts. This procedure brought to the selection of 2,109 pairwise comparisons in 717 GDS. In 1,876 out of 2,109 comparisons a "control" experimental factor was detectable thus allowing the definition of the sign of the altered expression measure (i.e. genes up- or down-regulated).

The calculation of the log fold change (LFC) and p value for each of the selected comparisons was performed with the linear regression algorithm implemented in the limma 3.10.3 R package [11]. The adjusted p values were obtained applying the Bayesian estimator implemented in the same package. The results of these analyses were used to initialize a PostgreSQL 9.1.3 relational database [12] that was used as data source for the CorrelaGenes application. The 2,188,704 probes obtained from different datasets were assigned to 190,155 unique identifiers. An automated procedure based on the biomaRt 2.10.0 R package [13] was developed to establish the relationship with NCBI official gene symbol and resulted in the unambiguous mapping of 35,968 identifiers. The remaining 154,284 identifiers were treated as spurious entities (i.e. corresponding to genomic elements no longer considered as transcribed); they cannot be used as target gene even if they were not discarded from the database. Despite the very limited number of corresponding probes (an average of 3.5 probes for unmapped identifiers against an average of 45.0 probes for official gene symbols) it is anyway possible that they would appear in the output list.

**Figure 3:**
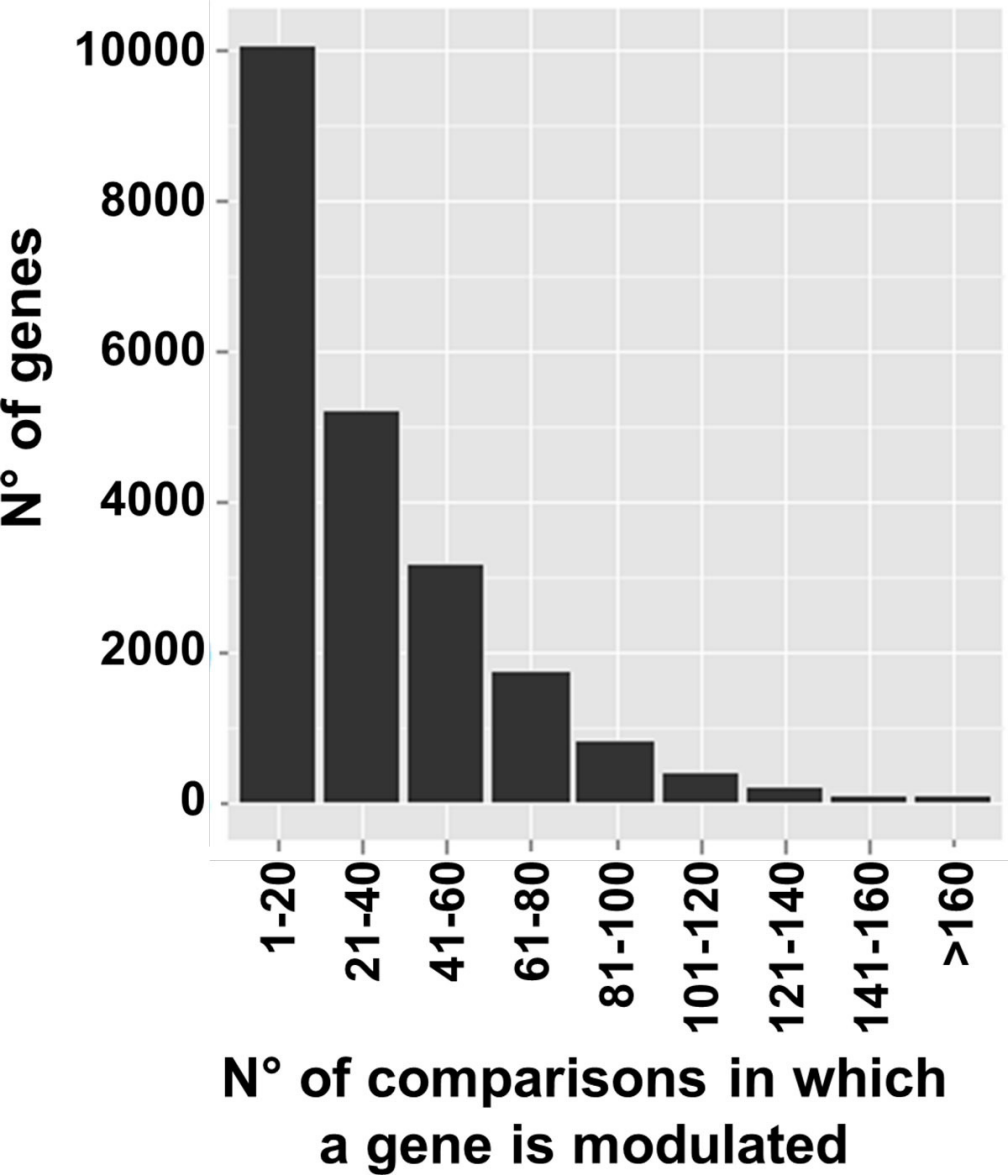
**Genes modulation in CorrelaGenes**. Histogram of the number of genes with respect to the number of comparisons in which they were found modulated.

To better characterize the dataset used by CorrelaGenes we analyzed the expression measures of the 35,968 official gene symbols that can be used as target genes. Defining a threshold of absolute LFC ≥ 1 and adjusted p value ≤ 0.05, a total of 14,163 genes were never found over- nor under-expressed. The remaining 21,805 showed modulation of their expression in an average of 19 comparisons. In Figure 3, the histogram of the distribution of the number of genes in relation to the number of comparisons in which they were found modulated is shown.

### ARM algorithm

In order to discover a set of genes that are frequently differentially co-expressed CorrelaGenes implements a customized version of an Association Rule Mining (ARM) algorithm. In the ARM formalism, datasets are organized in the form of transactions. Each transaction contains a list of elements, called items, whose nature depends on the application. In our context, each transaction corresponds to a comparison and includes the list of differentially expressed genes in that transaction. Such genes are selected on the basis of LFC and adjusted p value thresholds selected by the user. The application uses the transactions to identify association rules (ARs) of the form IF A THEN C (A=>C). In our context, these rules can be interpreted as: IF Set of Genes 1 is differentially expressed in an experiment THEN Set of Genes 2 is differentially expressed as well [14].

The ARM theory is based on the concept of frequent rule. A rule is frequent when it is verified by a sufficient amount of transactions in the dataset. In the case of gene expression experiments, an association is considered frequent if the involved genes are co-modulated in a sufficiently high number of comparisons. In the original ARM algorithm, the quality of an association is defined by the support. Support gives an idea of how frequent is a rule in a specific dataset. Given a dataset containing *N *transactions, the support of the rule A=>C will be calculated as *NA, C/N*, where *NA, C *is the number of transactions verifying the rule (i.e. containing all the items in A and C). An itemset is defined frequent if its support exceeds a user-defined support threshold.

In recent years different strategies were proposed to apply the ARM algorithm to the analysis of microarray expression data [15,16]. In this paper we use a simplified version of the ARM algorithm, which is based on two main points: (i) we look for associations containing only two genes and (ii) one of the involved genes is constrained to be the gene selected by the user (Target gene). In this way, the algorithm will look only for frequent item sets of cardinality 2 (i.e. 2 genes in the rule) and only for pairs of items involving the target gene as one of the members. Indicating with T the target gene and with X a generic gene in the dataset, we will thus look for rules of the kind IF T THEN X.

To measure the quality of the associations, we herein introduce the following indexes: co-presence, co-expression (support), Lift and χ^2 ^p value. These definitions are based on the concept of presence and modulation of a gene. A gene is defined present in a dataset if at least one of the probes corresponding to it was measured with an adjusted p value lower than the threshold fixed by the user. A gene is defined modulated if it is present in the dataset and its expression value exceeds the specified LFC threshold. The notations used in the definitions below are explained in Table 1.

**Table 1:** Description of the values used in the calculation of ARM indexes

Value	Description
N_Tp_	Number of comparisons where the target gene is present
N_Tp, Xp_	Number of comparisons where both the target gene and gene X are present
N_Tm, Xm_	Number of comparisons where the target gene and the gene X are both modulated
N_Tm, Xp_	Number of comparisons where the target gene is modulated and the gene X is present
N_Tp, Xm_	Number of comparisons where the target gene is present and the gene X is modulated

Co-presence is defined as the percentage of comparisons where both T and X are present.

$$co-pres=\frac{N_{Tp,Xp}}{N_{Tp}}$$

The co-pres index represents a technical parameter used to exclude from the analysis all the genes that were measured with the target in an insufficient number of times to allow a reliable estimation of the other ARM indexes.

Co-expression is defined as the percentage of comparisons where both gene T and gene X are differentially expressed. More formally:

$$co-expr=\frac{N_{Tm,Xm}}{N_{Tp,Xp}}$$

This is the adaptation of the notion of support for traditional ARM algorithm to our domain. The co-expr index can be used as a raw estimation of the biological relevance of the association of the gene X expression with the target. In our simulations it was insufficient to discriminate the biologically relevant associations from the background even if it can be used to further rank the output gene lists.

In traditional ARM algorithm, Lift is defined as the ratio between the confidence of a rule and the support of the consequent of the rule. For the scope of this paper, we define thus Lift as follow:

$$Lift=N_{Tp,Xp}\cdot \frac{N_{Tm,Xm}}{N_{Tp,Xm}\cdot N_{Tm,Xp}}$$

The Lift index was the first main parameter allowing the selection of the genes biologically related with the target. From its definition a Lift equal to one represents a gene randomly associated with the target while higher level of Lift identifies genes with a significant co-modulation. In our simulations a Lift threshold of two greatly improved the biological relevance of the output gene lists.

We perform a χ^2 ^test to evaluate the number of times the target gene and gene X result simultaneously differentially expressed compared to the expected value in the target population. The χ^2 ^p value is used to estimate the independence between the target and a gene X. Even if this type of index is rarely used to rank ARs, in our simulations, it resulted useful to discriminate the biologically relevant associations from the background.

Using ARM to mine frequently associated genes has several advantages. First of all the search procedure is very efficient as it is based on the Apriori principle. Second, it potentially allows a generalization towards considering any number of genes in the rule, naturally extending the present version of the method to more complex regulation scenarios.

### CorrelaGenes web interface

**Figure 4:**
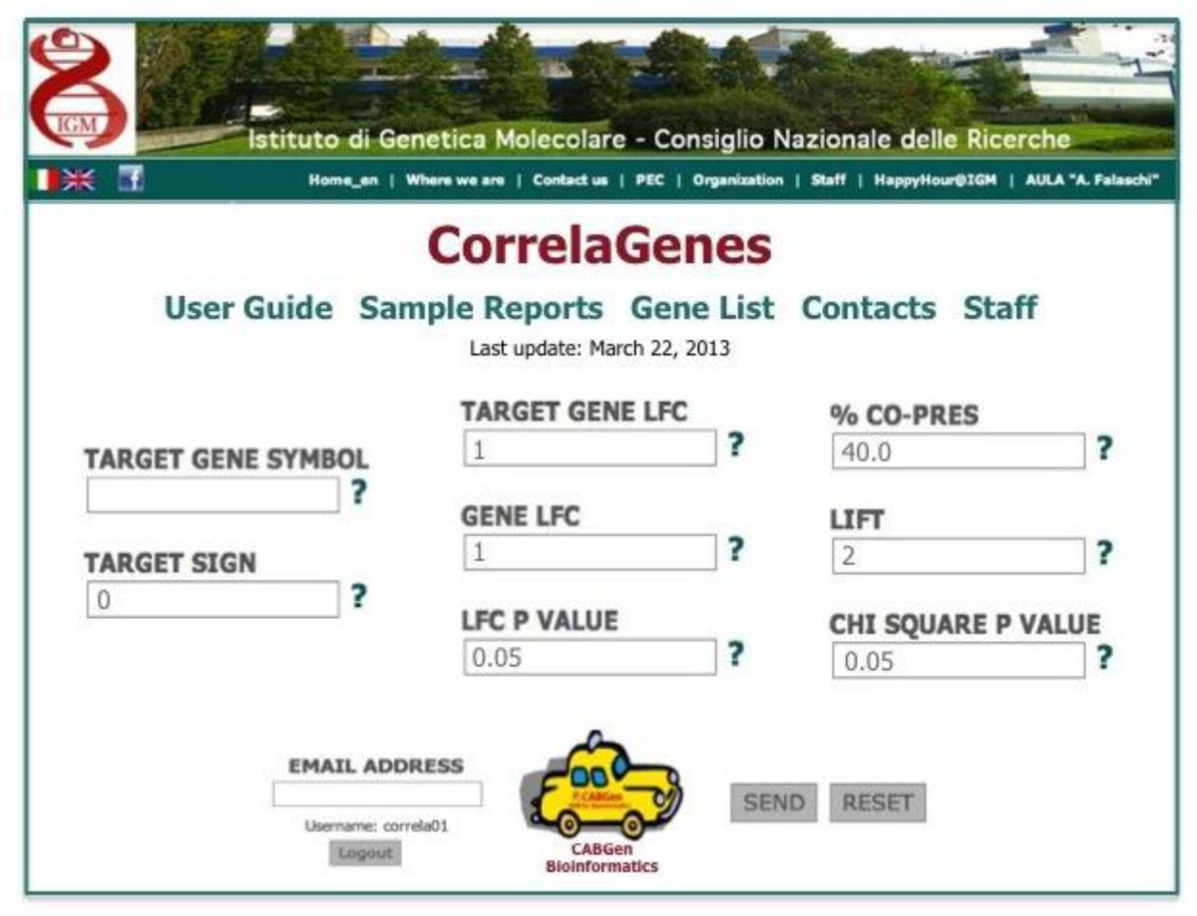
**Homepage of the CorrelaGenes web interface**.

A web interface was created to provide users with an easy and efficient access to the tool (Figure 4). Connecting to the CorrelaGenes web page [17], the user can start the analysis of the gene of interest by defining three sets of parameters: (i) the official symbol of the gene of interest ("Target Gene Symbol") and the sign of the differential expression (i.e. +1/-1 to restrict the analysis to the comparisons where the target gene appeared over/under expressed or 0 to consider both); (ii) the LFC (different values could be chosen for the "Target Gene LFC" and for "Genes LFC") and the adjusted p value thresholds for the definition of differentially expressed genes; (iii) the criteria to filter the list of related genes based on the ARM indexes ("% Co-Pres", "Lift", "χ^2 ^p value"). Flanking each input field a pop-up window is available to help users in the correct definition of parameters.

As the CorrelaGenes analyses are performed in batch an e-mail address is required to send the results back to the user that will initially receive a notification of the job submission followed by an email containing the link to the analysis output. The results are structured as tab-delimited text files and include an 8-rows header summarising all the analysis parameters and two additional rows indicating the number of comparisons where the target gene was found present or modulated. Below the header, a table reports the list of the related genes found together with their annotation details (i.e.: gene symbol, gene description, chromosome, cytogenetic band, strand, start position, end position and Ensembl ID) and all the ARM indexes calculated during the analysis (see Additional File 2).

### CorrelaGenes performances

The performance of the algorithm was evaluated in term of execution time. A preliminary analysis was performed with 100 randomly selected genes used as targets and with the following input values: "Target Sign" = 0, "Target Gene LFC" = 1, "Genes LFC" = 1, "LFC p value" = 0.05, "% Co-pres" = 40, "Lift" = 2 and "Chi-Square p value" = 0.05. Averaging on the considered 100 genes, the whole procedure requires a mean execution time of 190 seconds. We evaluated the average cost of each phase as percentage of the total execution time. The profiling of the code showed that the 50% of the total time is spent initializing the data, the 44% is spent creating the different gene lists and the 6% is actually spent generating the ARs.

## Results

### 100 genes simulation

To assess the tool functionality, 100 official gene symbols were randomly extracted and analyzed with CorrelaGenes. For the purpose of this simulation we extracted our sample among genes modulated in at least one comparison (i.e. absolute value of LFC ≥ 1, adjusted p value ≤ 0.05). We run all the analysis setting the following parameters: "Target Sign" = 0, "Target Gene LFC" = 1, "Genes LFC" = 1, "LFC p value" = 0.05.

A threshold of "% co-pres" = 40 was applied to limit the number of false positive results due to genes co-measured in a small number of comparisons. The output lists obtained included an average of 4,403 genes (range 262-13,170, median = 3,727). Additional File 3 shows the impact of different thresholds of the co-pres index on the total number of related genes.

**Figure 5:**
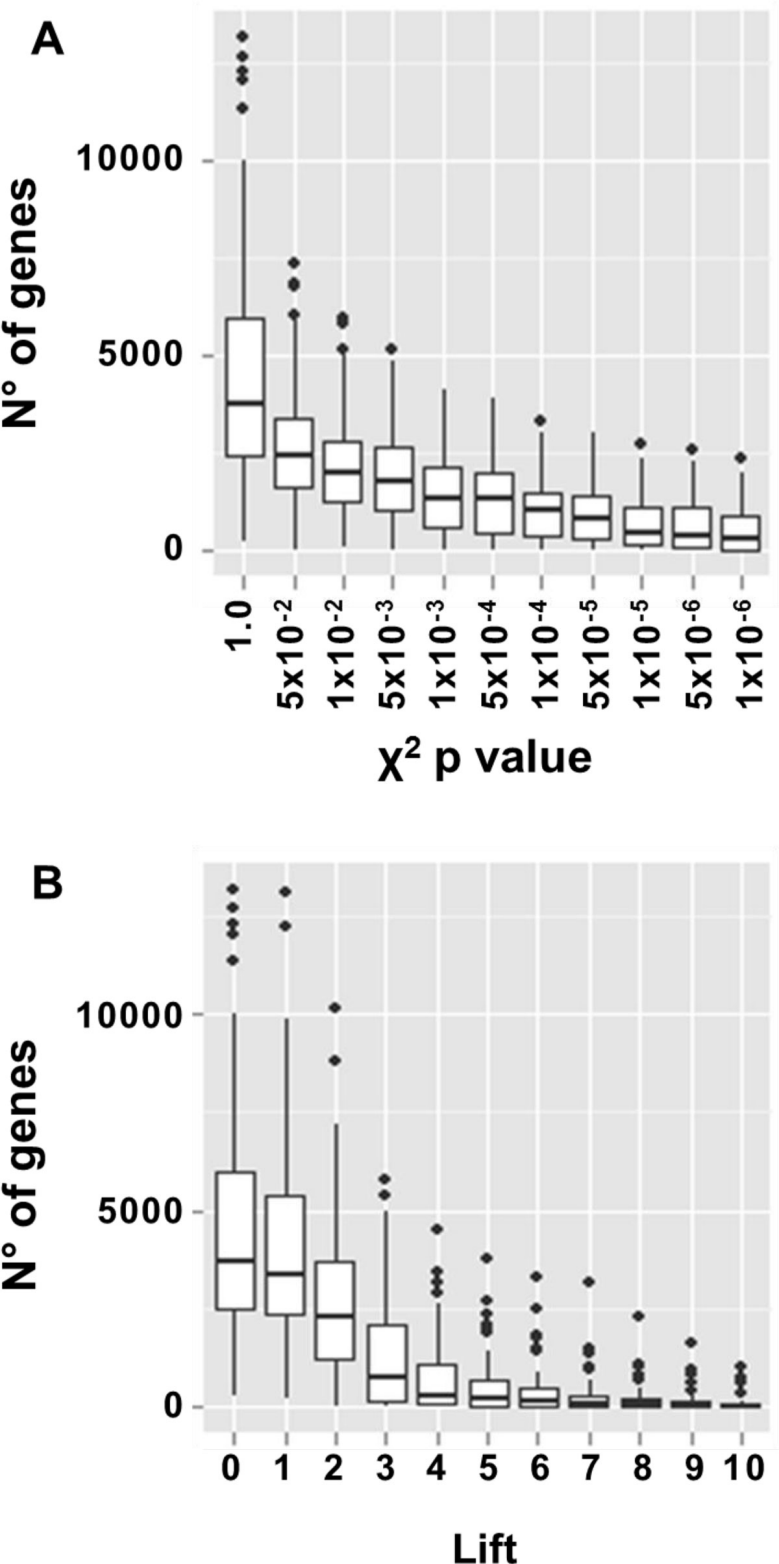
**Impact of the ARM indexes on the number of genes in the output lists**. (A) Box-plot of the number of genes with respect to different thresholds of χ^2 ^p value. (B) Box-plot of the number of genes with respect to different thresholds of Lift.

Different thresholds of the χ^2 ^p value and of the Lift indexes were evaluated (Figure 5). Increasing by a factor of 10 the threshold of the χ^2 ^p value starting from 0.05 and 0.01 resulted in an almost linear reduction of the number of related genes (Figure 5A). On the contrary, even small increases in the Lift index drastically reduced the number of genes in the output lists (Figure 5B). Increasing the Lift index from 1 to 4 resulted in halving the number of related genes while for Lift values greater than 5 the median number of selected genes is always below 40. A box-plot showing the combined effect of χ^2 ^p value and Lift indexes is presented in Additional File 4.

### Biological relevance of the results: analysis of the *PRPF19 *gene

**Figure 6:**
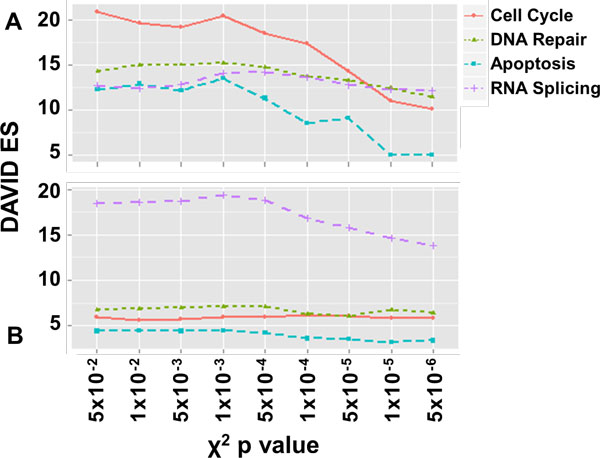
**Analysis of the *PRPF19 *gene lists**. Trend of the DAVID Enrichment Scores (ES) with respect to different thresholds of χ^2 ^p value with (A) % co-pres = 40 and (B) % co-pres = 40 and Lift = 2 (the GO terms list with related Benjamini p value is available in Additional Files 7).

To assess the biological relevance of the CorrelaGenes output gene lists we chose to analyze the list obtained using *PRPF19 *as Target gene (see Additional File 5). *PRPF19 *is a well-characterized gene whose product plays a role in the formation of the RNA splicing complex [18] and it is also described to be involved in DNA repair [19], in the regulation of cell cycle [20] and apoptosis [21]. The output lists, obtained using the same expression thresholds set for the simulation study and comparing different thresholds of Lift and χ^2 ^p value, were evaluated through the Database for Annotation, Visualization and Integrated Discovery (DAVID v6.7) [22] Functional Annotation Clustering using Gene Ontology (GO) biological process as source of information [23]. Applying different cut-offs for the χ^2 ^p value (from 0.05 to 5 × 10^-6^) we obtained a relatively small decrease in the total number of extracted genes (from 2,526 to 1,406). The analysis of the nine gene lists showed the presence of clusters (Figure 6A) related to RNA splicing, cell cycle, DNA repair processes all with a DAVID Enrichment Scores (ES) always greater than 10.0. Clusters related to apoptosis showed the same level of ES even if they strongly decreased at χ^2 ^p value threshold smaller than 5 × 10^-4^. The analysis performed with a Lift threshold of 2 generated a list of 1,721 genes that resulted mainly enriched in clusters related to RNA splicing (ES = 18.4) whereas other processes showed ES between 4.0 and 6.0. The analysis performed with a Lift threshold of 3 resulted in a gene list containing only 321 genes with lesser significant DAVID annotations (see Additional File 6). We repeated the χ^2 ^p value analysis with a threshold of Lift = 2 obtaining lists including 1,720 to 1,189 genes. The DAVID Functional Annotation Clustering highlighted RNA splicing as the most enriched cluster with an ES of 18.4 (Figure 6B) while the other clusters obtained lower enrichment scores: DNA repair (ES = 6.8), cell cycle (ES = 5.9) and regulation of apoptosis (ES = 4.4).

**Figure 7:**
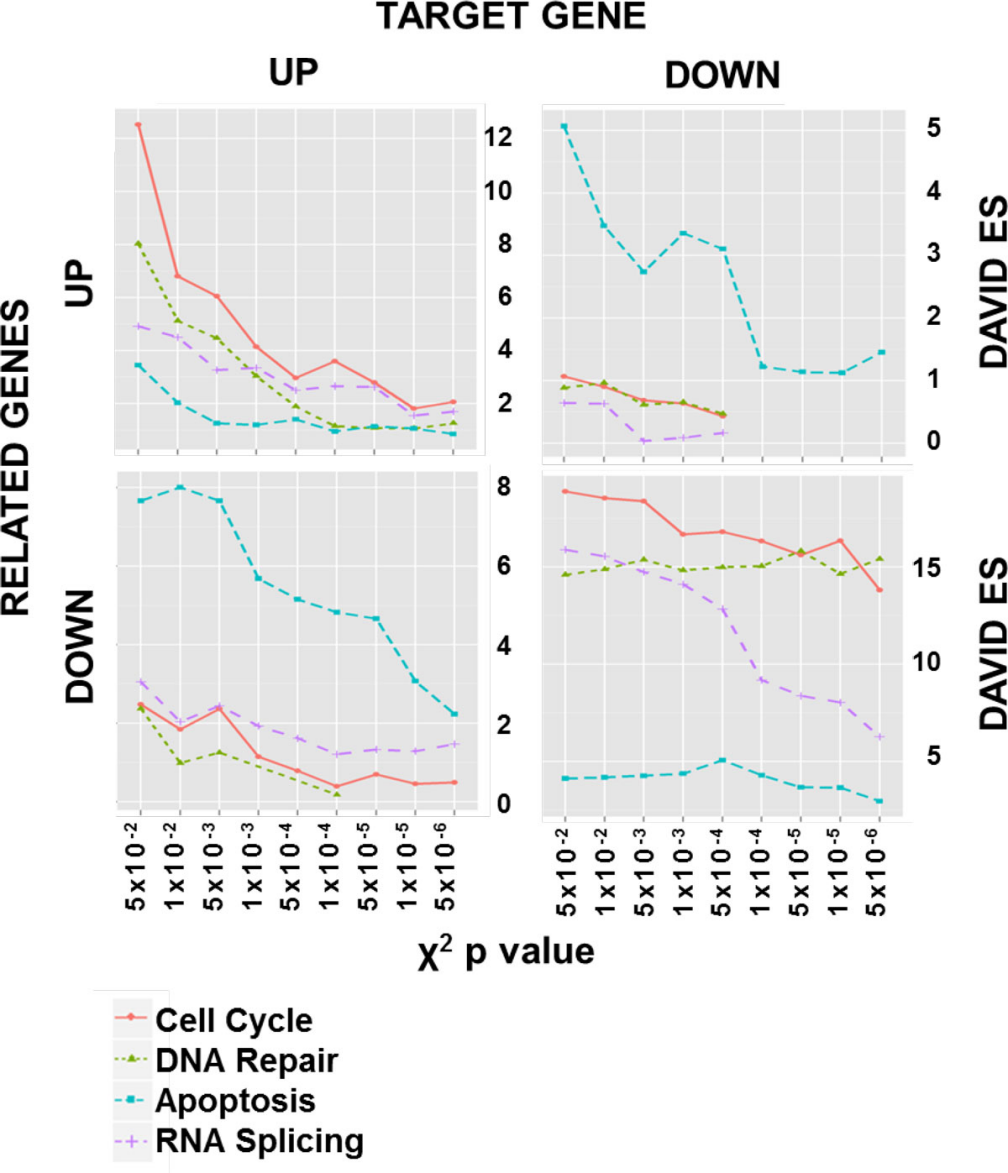
**Analysis of up- and down-regulation in *PRPF19 *gene lists**. Trend of the DAVID Enrichment Scores (ES) with respect to different thresholds of χ^2 ^p value with % co-pres = 40 and Lift = 2 distinguishing between up- or down-regulated target and related genes (the GO terms list with related Benjamini p value is available in Additional File 7).

To further characterize *PRPF19 *related genes we performed the analysis distinguishing between up- or down-regulation of the target and of related genes. The results of this analysis (Figure 7) allowed to identify different expression relationships between the target and its correlated genes. In particular, the apoptotic process appeared inversely related to the up- or down-regulation of *PRPF19 *while DNA repair and cell cycle appeared directly related. Finally, the expression level of the *PRPF19 *gene seemed to be a limiting factor for the RNA splicing process as its down-regulation results in a list of down-regulated genes highly enriched (ES = 15.9) for this function.

## Discussion

In this paper we described the development of a new bioinformatics tool, CorrelaGenes, which exploits publicly available expression data to extract lists of genes transcriptionally correlated with a gene of interest.

The transcriptional profiles used by CorrelaGenes came from 978 human datasets obtained from the GEO archive that were manually elaborated to select a subset of 2,109 comparisons with a consistent biological rationale. This represents an added value of this new tool as the automated procedures, often employed to analyze the GEO data, introduce distortions due to the presence of spurious correlations.

Moreover, due to the increasing number of curated datasets available at the GEO archive, we planned a quarterly update of CorrelaGenes. This process will include the addition of the new studies along with the manually curated knowledge-based selection of pairwise comparisons thus improving the overall accuracy of the results.

The CorrelaGenes tool was implemented with a customized ARM algorithm that provides the user with a variety of indexes through which it is possible to modulate the features of the extracted gene list thus exploring different biological perspectives when considering the function of the target gene. The possibility of choosing different levels of gene expression modulations (i.e. values of LFC) and distinguishing between over- or under-expression of the target gene (i.e. Target sign) allowed to focus the analysis on specific aspects of cell function and regulation. The tuning of the "co-pres" filter appeared particularly helpful in the reduction of experimental background as it limits the analysis to a number of observations suitable to avoid the majority of false positive results. Moreover, combinations of the Lift and χ^2 ^p value can be used to dissect pathways where a target gene acts in different cellular conditions thus helping to untangle its complex functioning. The values we suggested as default appeared suitable for a general characterization of a gene of interest. Depending on the user expectations, more stringent thresholds (i.e. higher Lift and lower χ^2 ^p value) could be used to reduce the occurrence of false positive results being aware that a concomitant reduction in informative genes could not be ruled out.

The availability of large amount of data boosted the development of new bioinformatics tools allowing to explore many aspects of gene expression profiles applying different types of meta-analysis. To create CorrelaGenes we focused on two aspects that we considered not fully exploited: the possibility to combine many studies with no restriction in the platform selection and with no restrictions in the experimental factors. The first point guided us to the choice of the ARM algorithm that we found to be robust handling the high number of missing values present in data source. To address the second point, we included in CorrelaGenes all curated datasets available in GEO taking in consideration all different experimental factors used to perturb cell physiology. This feature might allow CorrelaGenes to explore different aspects of gene functions and represents the main difference with respect to other tools as Oncomine and PubliME that focus their analysis on cancer biology.

The gene lists obtained with our tool can be analyzed by other bioinformatics resources to get insight into the biological processes or molecular mechanisms where the genes appeared involved. In this paper we used the DAVID web tool to annotate the gene list corresponding to the *PRPF19 *target gene. As illustrated in the results section, the obtained list is consistent with the known biological role of this gene thus providing a reliable source where to look for new players of the same process or to search for unknown processes in which the target gene could be involved.

## Conclusions

The CorrelaGenes tool, through a new approach for the characterization of human genes transcriptional profiles, could contribute to the comprehension of molecular pathways regulating cell physiology.

## Availability and requirements

• **Project name: **CorrelaGenes

• **Project home page: **http://www.igm.cnr.it/cabgen/

• **Operating system(s): **Platform independent

• **Software requirements: **Web browser (supported browser Firefox, Safari, Chrome)

• **Other requirements: **an e-mail account

• **Programming language: **Fortran, R 2.14, PHP5.2.6, TYPO3 4.5.6

• **Updates: **the tool will be updated quarterly

• **Licence: **Free to academic, government and non-profit users for non-commercial use.

## List of abbreviations

ARM: Association Rule Mining; ARs: Association Rules; DAVID: Database for Annotation, Visualization and Integrated Discovery; EBI: The European Bioinformatics Institute; ES: Enrichment Scores; GDS: GEO DataSets; GEO: Gene Expression Omnibus; GO: Gene Ontology; LFC: Log Fold Change; MIAME: Minimum Information About a Microarray Experiment; NCBI: National Center for Biotechnology Information.

## Competing interests

The authors declare that they have no competing interests.

## Authors' contributions

PC, SB, AM and GB conceived this research. SB did the coordination of the study. PC and SB performed the data pre-processing procedures and the manual selection of comparisons. PC, LS, SB and GS designed the ARM algorithm. SR implemented and optimized the algorithm. AL and FC developed the web interface. PC and SR did the simulation analysis. PC, SB, AM and GB analyzed biological results. PC, SR, LS, GS and SB wrote the manuscript. All authors read and approved the final manuscript.

## Supplementary Material

Additional file 1 **Contrast matrix creation and comparisons selection**. The file includes, in PDF format, the detailed description of the contrast matrix creation process and the curated knowledge-based procedure workflow. Click here for file

Additional file 2 **Description of the output file header**. Click here for file

Additional file 3 **Impact of the % co-pres index on the number of genes in the output lists**. Click here for file

Additional file 4 **Impact of the Lift and χ^2 ^p value indexes on the number of correlated genes**. Click here for file

Additional file 5 ***PRPF19 *Output gene list**. The file include the output gene list, in a tab-delimited text format, obtained by the analysis of the *PRPF19 *gene. Click here for file

Additional file 6 **Analysis of the PRPF19 gene lists**. Trend of the DAVID Enrichment Scores (ES) respect to different Lift thresholds. Click here for file

Additional file 7 **DAVID Functional Annotation Chart related to the output lists used in **Figures 6, 7. Click here for file

## Declarations

All the publication costs were supported by Cariplo Foundation grant n. 2010/0253, and by Progetto di interesse CNR-MIUR "Invecchiamento" and by IMATI-CNR of Pavia.

This article has been published as part of *BMC Bioinformatics *Volume 15 Supplement 1, 2014: Integrated Bio-Search: Selected Works from the 12th International Workshop on Network Tools and Applications in Biology (NETTAB 2012). The full contents of the supplement are available online at http://www.biomedcentral.com/bmcbioinformatics/supplements/15/S1.
