# Supplementary material for: CorrelaGenes: a new tool for the interpretation of the human transcriptome
Source: BMC Bioinformatics. 2014 Jan 10;15(Suppl 1):S6. doi: 10.1186/1471-2105-15-S1-S6 (PMC4016313; doi:10.1186/1471-2105-15-S1-S6)
Supplement: Additional file 1 — Contrast matrix creation and comparisons selection. The file includes, in PDF format, the detailed description of the contrast matrix creation process and the curated knowledge-based procedure workflow. [file 1471-2105-15-S1-S6-S1.pdf]

DataSet GDS2516 from the GEO Web site

Graphical representation of the GDS2516 experimental design

Box plot of the value distribution related to each sample

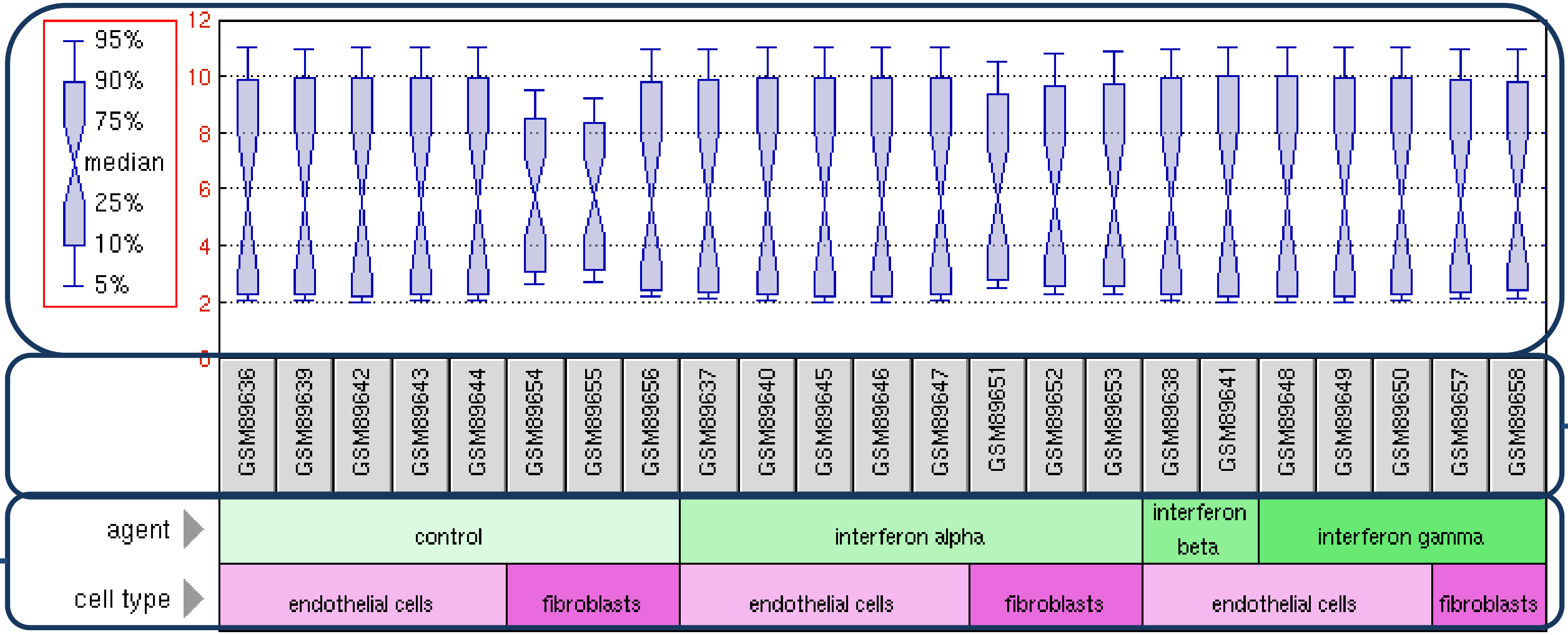

Experimental design

Sample identification

Contrast matrix creation and comparisons selection

Step 1: sample groups identification

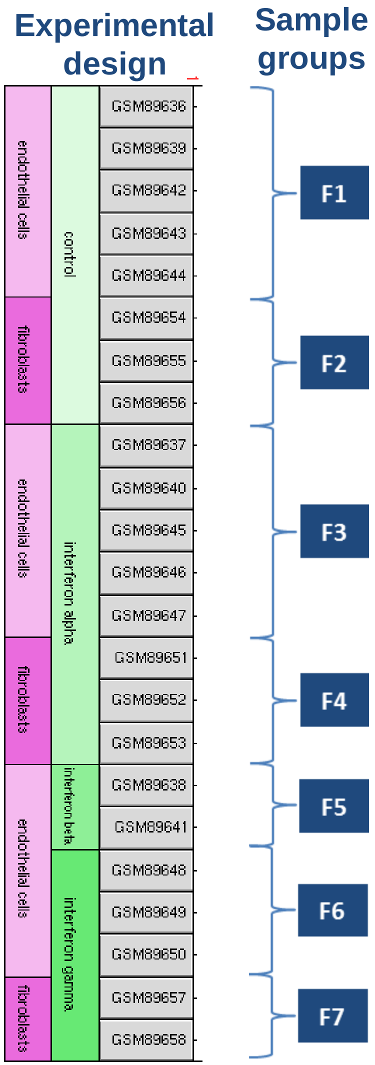

The experimental design obtained from the microarray meta-data is used to group all the samples that share the same experimental conditions.

From the GDS2516 experimental design represented on the right we have identified seven different groups of samples:

F1 - Endothelial cells: control
F2 - Fibroblasts: control
F3 - Endothelial cells: interferon alpha
F4 - Fibroblasts: interferon alpha
F5 - Endothelial cells: interferon beta
F6 - Endothelial cells: interferon gamma
F7 - Fibroblasts : interferon gamma

Contrast matrix creation and comparisons selection

Step 2: contrast matrix creation

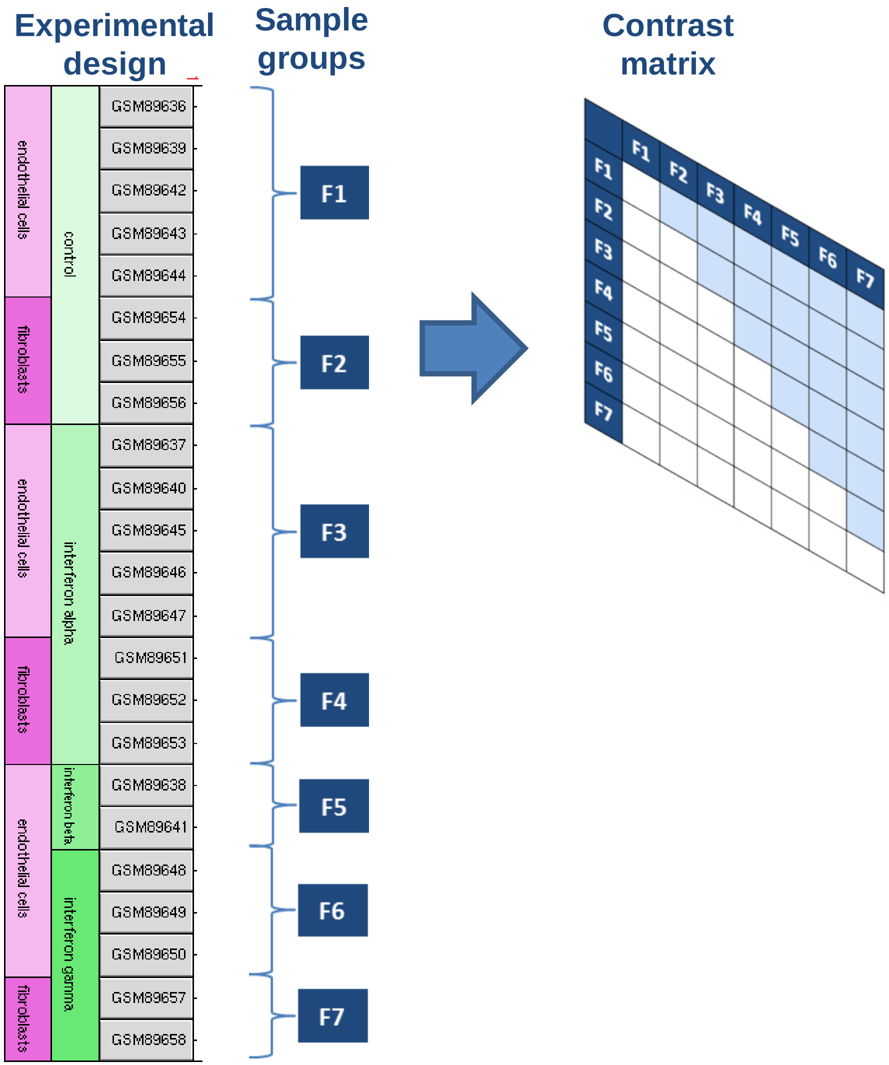

To identify all the possible pairwise comparisons we have automatically created a table, called contrast matrix, including all the possible combinations among the different sample groups.

For the GDS2516 the contrast matrix allowed to identify 21 possible pairwise comparisons

Contrast matrix creation and comparisons selection

Step 3: manual selection of the pairwise comparisons

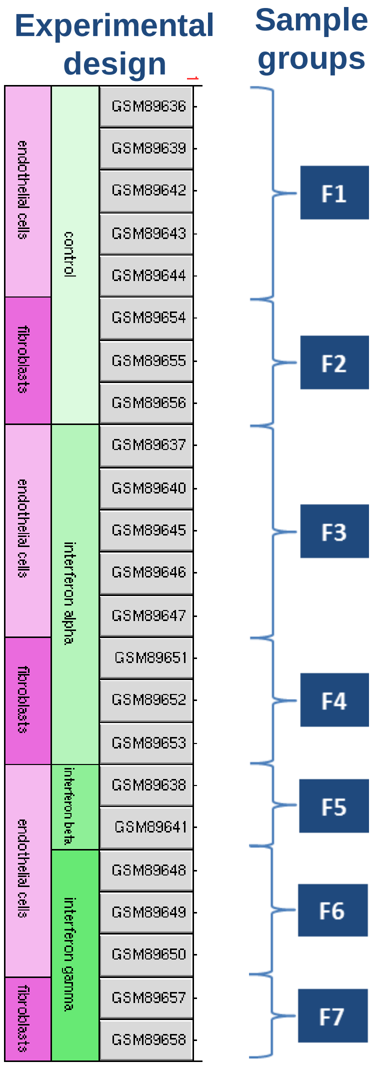

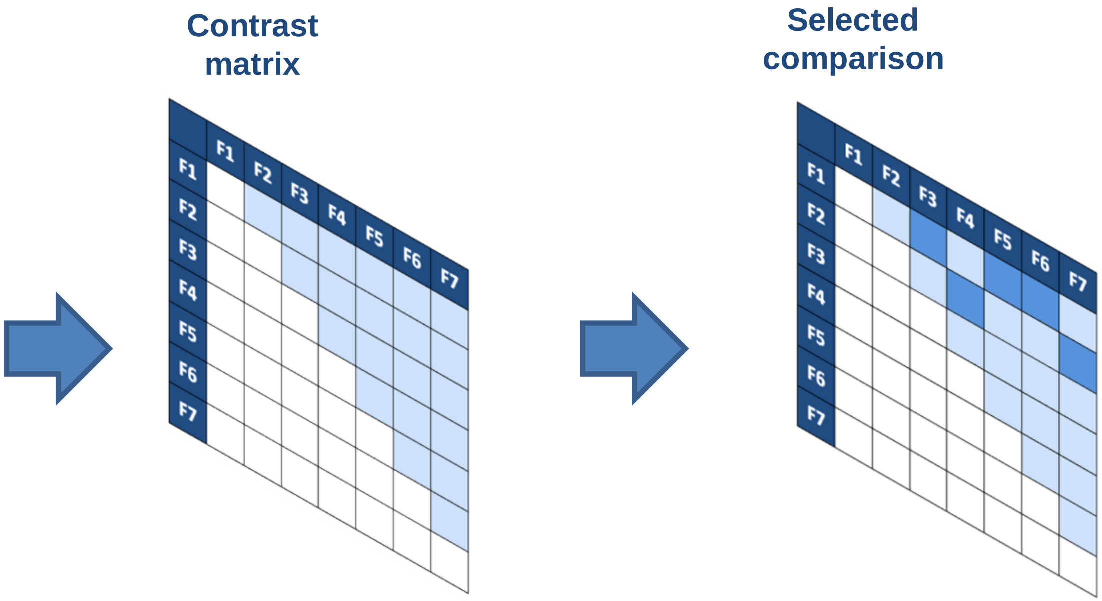

Not all the pairwise comparisons identified by the contrast matrix have a clear biological meaning.

For this reason we defined a manually curated knowledge-based procedure to discard all the comparisons lacking a clear experimental rationale.

In the case of the GDS2516 only five of the 21 possible pairwise comparisons satisfied our selection criteria.

Contrast matrix creation and comparisons selection

Step 4: the manually curated knowledge-based procedure

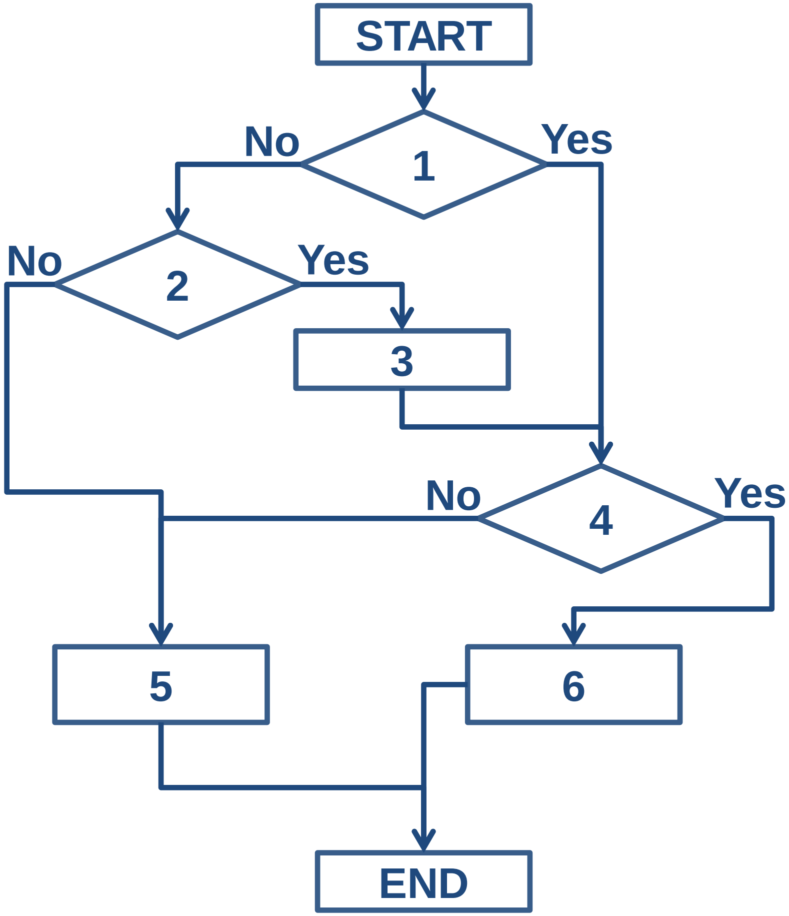

1. Is there only one cell line involved in the experiment?
2. There are experimental factors other than the cell lines?
3. The experimental design is split to compare only the sample groups based on the same cell line exposed to different experimental factor
4. Is it possible to identify a sample group to be considered as «Control»?
   We considered as control:
   - the lower time among different time points (usually the time point 0)
   - the lower treatment dosage (usually the untreated sample)
   - healthy tissues compared with different disease state
5. We selected all the comparisons among the different experimental groups
6. We selected all the comparisons including the sample group identified as «Control»
