# Supplementary material for: CorrelaGenes: a new tool for the interpretation of the human transcriptome
Source: BMC Bioinformatics. 2014 Jan 10;15(Suppl 1):S6. doi: 10.1186/1471-2105-15-S1-S6 (PMC4016313; doi:10.1186/1471-2105-15-S1-S6)
Supplement: Additional file 2 — Description of the output file header. [file 1471-2105-15-S1-S6-S2.pdf]

Additional File 2: Description of the output file header

**Value**	**Description**
id_gene_symbol	Related gene official gene symbol.
description	Related gene description.
chromosome_name	Chormosome.
band	Cytogenetic band.
strand	Strand.
start_position	Related gene start position .
end_position	Related gene end position.
id_ensembl	Ensembl identifier.
sign	Related gene LFC sign ("o" when the analysis was run with sign "0"; when the analysis was run with +1/-1 the sign can be "+" for up-regulation or "-" for down-regulation).<br>Note: when an analysis was run with sign the same gene could appear in the list two times.
GTpGXp	Number of comparisons where the target gene and the related genes are presents.
GTmGXm	Number of comparisons where the target gene and the related genes are modulated.
GTnGXn	Number of comparisons where the target gene and the related genes are NOT modulated.
GTnGXm	Number of comparisons where the target gene is NOT modulated while the related gene is modulated.
GTmGXn	Number of comparisons where the target gene is modulated while the related gene is NOT modulated.
copres	% of co-pres index.
coexpr	% of co-expr index.
lift	Lift index.
chi	X^2 p value index.
