# Supplementary figures and images for: CorrelaGenes: a new tool for the interpretation of the human transcriptome
Source: BMC Bioinformatics. 2014 Jan 10;15(Suppl 1):S6. doi: 10.1186/1471-2105-15-S1-S6 (PMC4016313; doi:10.1186/1471-2105-15-S1-S6)

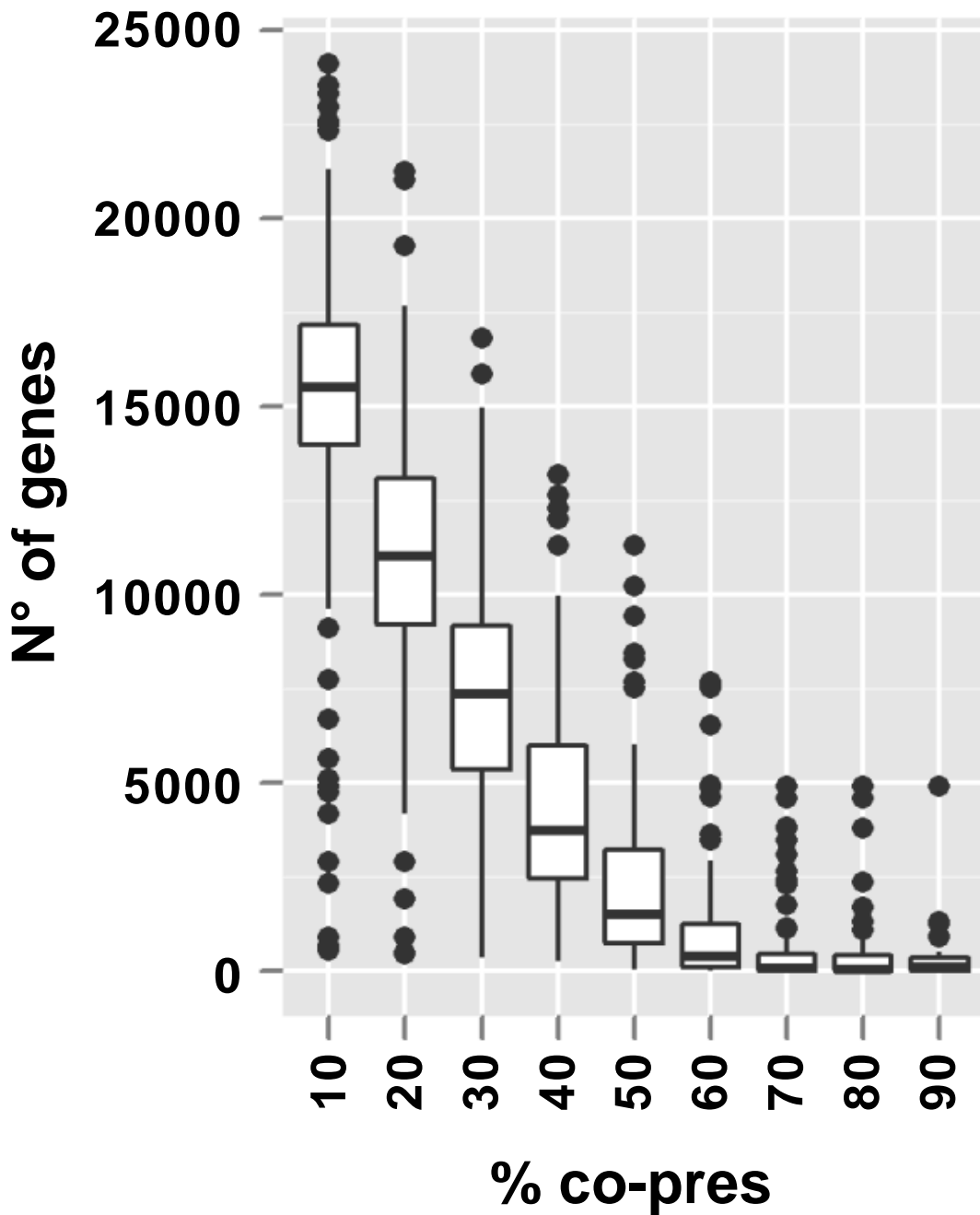

**Additional file 3: Impact of the % co-pres index on the number of genes in the output lists**

Supplement: Additional file 3 — Impact of the % co-pres index on the number of genes in the output lists. [file 1471-2105-15-S1-S6-S3.pdf]

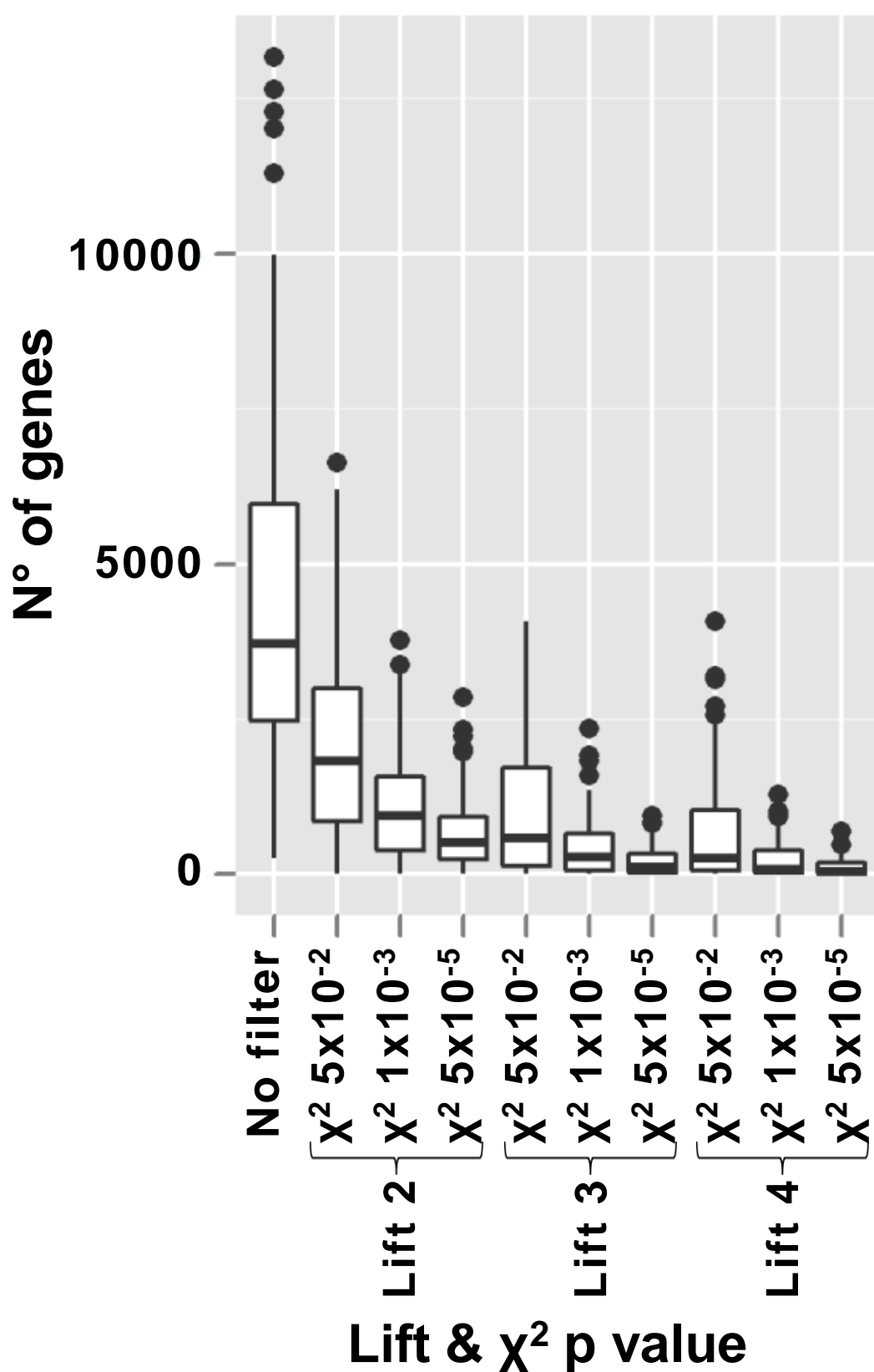

**Additional file 4: Impact of the Lift and  $\chi^2$  p value indexes on the number of correlated genes**

Supplement: Additional file 4 — Impact of the Lift and χ2 p value indexes on the number of correlated genes. [file 1471-2105-15-S1-S6-S4.pdf]
