# Supplementary material for: CorrelaGenes: a new tool for the interpretation of the human transcriptome
Source: BMC Bioinformatics. 2014 Jan 10;15(Suppl 1):S6. doi: 10.1186/1471-2105-15-S1-S6 (PMC4016313; doi:10.1186/1471-2105-15-S1-S6)
Supplement: Additional file 6 — Analysis of the PRPF19 gene lists. Trend of the DAVID Enrichment Scores (ES) respect to different Lift thresholds. [file 1471-2105-15-S1-S6-S6.pdf]

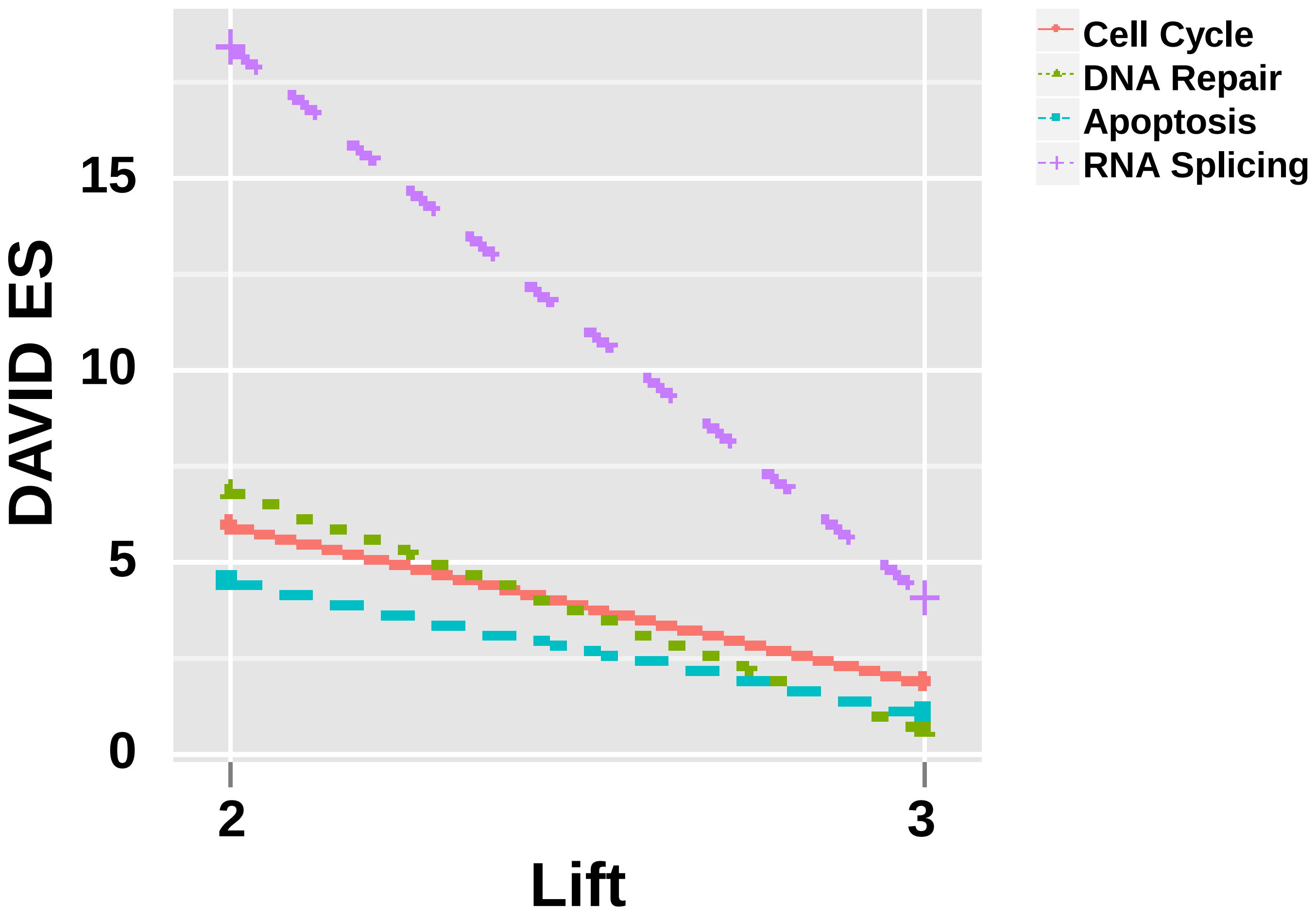

Additional file 6: Analysis of the PRPF19 gene lists

Trend of the DAVID Enrichment Scores (ES) respect to different Lift thresholds.
